# Supplementary material for: Memory effects of Eurasian land processes cause enhanced cooling in response to sea ice loss
Source: Nat Commun. 2019 Nov 8;10:5111. doi: 10.1038/s41467-019-13124-2 (PMC6841963; doi:10.1038/s41467-019-13124-2)
Supplement: Supplementary file 1 — Supplementary Information [file 41467_2019_13124_MOESM1_ESM.pdf]

Supplementary Information

# Memory effects of Eurasian land processes cause enhanced cooling in response to sea ice loss

**Nakamura *et al.***

**Supplementary Note 1.**

**Nonlinear response depending on the initial land condition.** There is a strong nonlinearity in the responses between the initial land surface conditions. Strong and obvious negative AO-like circulation patterns and mid-latitude cooling anomalies are found in the memory effect with LICE's land condition (Memory [LICE–LICE†], right panels of Supplementary Figure 1a and 1c) and also in the sea ice effect with LICE's land condition (Sea ice [LICE†–HICE], left panels of Supplementary Figure 1b and 1d).

Interestingly, significant anomalies only appear when the experiments are performed with the LICE land condition. The atmosphere and land conditions adjusted to the high sea ice condition appear to be more stable with respect to the sea ice change than those adjusted to the low ice condition. To shift the climate regime from one adjusted to the high ice condition to one adjusted to the low ice condition might require repeated forcing from persistently low sea ice conditions, as have been observed in recent decades.

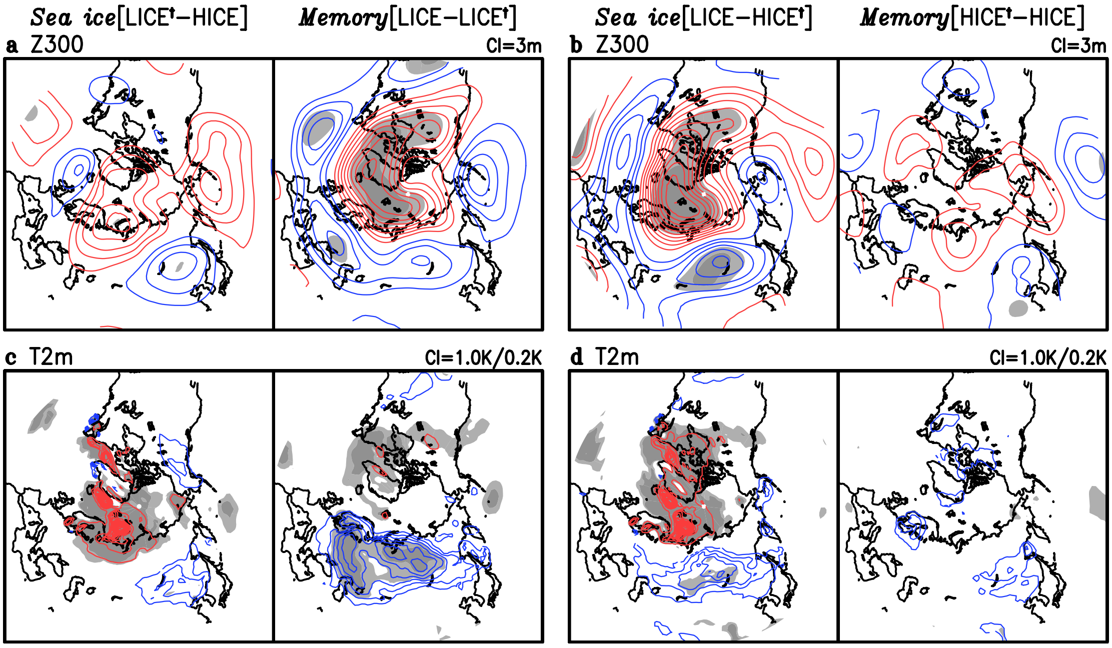


**Supplementary Figure 1. Simulated winter atmospheric responses to sea ice reduction.** This is the same as Fig. 2 in the main text except that the Sea ice and Memory effects are separately evaluated by the procedure using **a** and **c** LICE† and **b** and **d** HICE† runs, corresponding to the left and right panels in Fig. 1, respectively. Red and blue indicate positive and negative anomalies, respectively, and light and heavy grey shadings indicate statistical significance exceeding 95% and 99%, respectively.

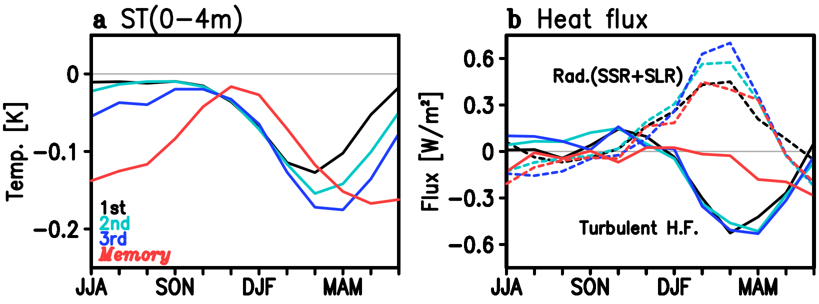


**Supplementary Figure 2. Development of Sea ice and Memory effects in the soil temperature.** Seasonal evolutions of soil temperature anomalies and heat flux anomalies averaged over Siberia/East Asia (90–120°E, 40–60°N). **a** Soil temperature 0–4 m depth and **b** surface heat flux due to turbulent heat flux (solid lines) and surface radiation flux (sum of short-wave and long-wave radiation, dashed lines) are shown. For the heat flux anomalies, a positive anomaly indicates an increase of upward flux. Black, light blue, and blue colours indicate Sea ice effects from the first, second, and third year of the integration of the initialized experiment. Red indicates the Memory effect.

**Supplementary Note 2.**

**Characteristics of the seasonal cycle of soil conditions to form the memory effect.** We performed additional 'initialized run' experiments that extended the integration period by three years. Specifically, we examined the characteristics of the memory effect, particularly the way in which they evolve beyond interannual cycles.

As we discuss in the main text, circulation anomalies due to the sea ice effect bring cold anomalies over Siberia during winter. This Siberian cooling forces the soil to cool in the late winter of every year (Supplementary Figure 2a). Cold soil temperature anomalies intensify year by year, and the coldness peak moves later each year (see Fig. 6 in the main text). The largest difference between the sea ice and memory effects is anomalous soil coldness in the late spring to early autumn.

Next, we examined the causes of this soil coldness. In the sea ice effect, during late winter to early spring, anomalous soil coldness is largely led by the anomalous upward radiation forcing (Supplementary Figure 2b, dashed lines). This corresponds to a shortage of short-wave radiation due to the increased snow cover (Fig. 5 in the main text) because the contribution of long-wave radiation is small (not shown). In turn, anomalous soil coldness cools the atmosphere through turbulent heat flux anomalies (Supplementary Figure 2b, solid lines). In the memory effect, as a result of the delayed coldness peak, the anomalous soil coldness cools the atmosphere through turbulent heat flux during late spring. After that, heat flux anomalies are nearly zero during summer to autumn, while the anomalous coldness gradually weakens. This indicates that the air temperature that adjusted to the cold ground condition in spring persists until autumn (Fig. 2c and 2d in the main text). This, in turn, possibly brings anomalous coldness and early snowfall in the autumn and early winter as a memory effect (Fig. 5 in the main text).

**Supplementary Note 3.**

**Relationship between ground conditions and the Eurasian winter climate in AMIP-like simulations.** We analysed the output of the historical simulation using AFES ([1]Ogawa *et al.*, 2018). There are two types of 30-member ensemble simulations for the 1979–2014 period. One is a historical simulation forced by historical monthly mean SST and sea ice boundary conditions based on the Merged Hadley/OI SST dataset ([2]Harrell *et al.*, 2008). The other is similar to the historical simulation, but SST was fixed as the climatological mean annual cycle. Details of the simulation configuration are described by [1]Ogawa *et al.* (2018). Here, we evaluate the relationship between long-term trends of preceding ground conditions (October-November snow cover and July-August-September soil temperature) and winter (December-January-February) surface temperature averaged over the eastern part of the Eurasian continent (60–120°E, 40–60°N). In this historical simulation using AGCM, the long-term trend varies among ensemble members. Such intra-ensemble differences could occur due to random noise from the atmospheric internal variation or variation of ground conditions. We then evaluate the intra-ensemble relationship.

Autumn snow cover seems to have no relation to Siberian/East Asian winter temperature in the historical simulation (Supplementary Figure 2a, Varying SST&ICE); the correlation coefficient is nearly zero. In the climatology-SST simulation, this

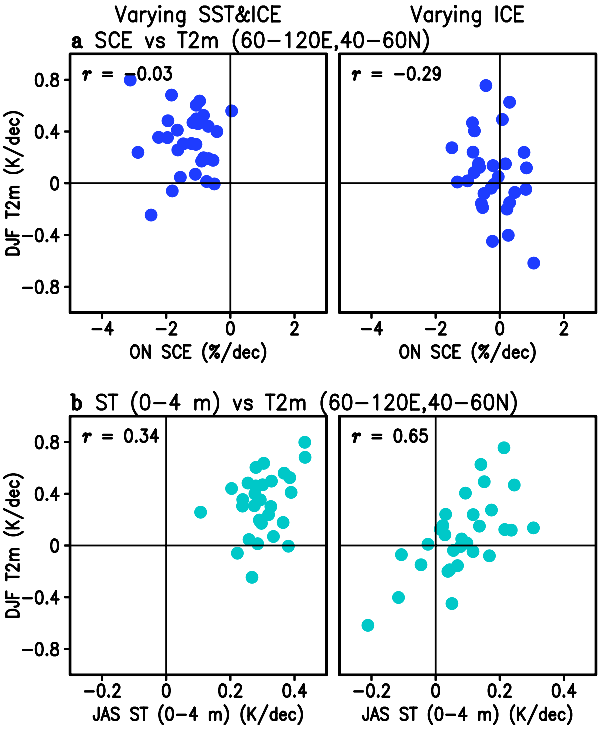


**Supplementary Figure 3. Intra-ensemble relationship between preceding ground conditions and winter Siberian/East Asian climate.** Scatter plots of the long-term trends of ground conditions and winter temperature at 2 m height (T2m) averaged over the eastern part of the Eurasian continent (60–120°E, 40–60°N). Ground conditions of **a** October-November snow cover extent (SCE) and **b** July-August-September soil temperature (ST) at 0–4 m depth are evaluated. Each point indicates the result from the individual 30 ensemble members. Correlation coefficients among ensemble members are given in each panel.

correlation becomes more negative, indicating that more snow brings a colder winter (Supplementary Figure 2a, Varying ICE). Summer soil temperature has a positive and higher correlation: 0.34 in the historical simulation and 0.65 in the SST-fixed simulation (Supplementary Figure 2b). This indicates that colder soil conditions in summer bring a colder winter.

These results suggest that the ground conditions (autumn snow and summer soil temperature) have the potential to affect the winter climate even in the long term. However, other external conditions, such as SST variations, largely disturb this relationship because the relationship is stronger in varying ICE than in varying SST&ICE.

## Supplementary Note 4.

**Observational evidence of the relationship between sea ice variation and ground temperature anomalies.** To evaluate observation-based evidence supporting our simulation results, we conducted an additional analysis using borehole temperature data from the GTN-P dataset ([3]Biskaborn *et al.*, 2019). Although

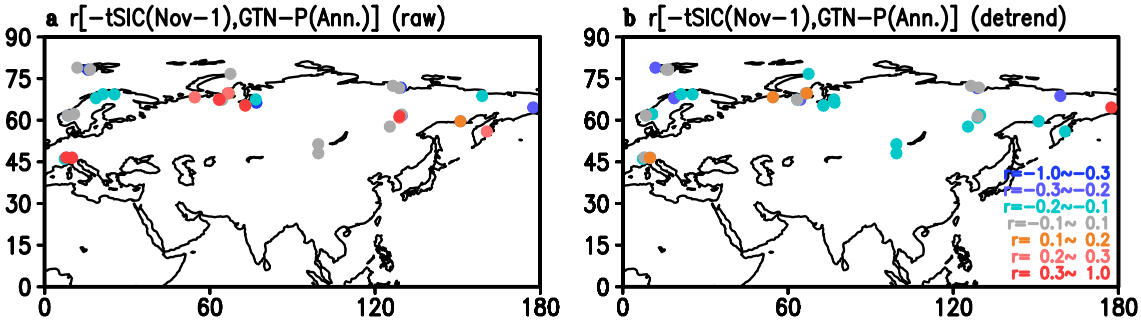


**Supplementary Figure 4. Observation-based relationship between sea ice variation and ground temperature anomalies.** Correlation coefficients between the sea ice concentration over the Barents/Kara Seas (30–90°E, 65–85°N) in the previous November and annual mean borehole temperature data from the GTN-P dataset ([3]Biskaborn *et al.*, 2019). All stations in the eastern Northern Hemisphere are used except for stations with less than 5 years of available data years or with elevations above 3000 m. The calculation period is 10 years based on the GTN-P data period of 2007–2016. Correlation coefficients at each GTN-P station calculated **a** without and **b** with detrending are shown. Note that the sign of coefficient is reversed to show anomalies associated with a decrease in sea ice.

the data period is 10 years, these *in-situ* observations are quite helpful in examining the role of ground processes. We calculated the correlation between preceding sea ice anomalies over the Barents/Kara Seas and annual mean borehole temperatures at individual stations.

Correlations are generally positive, indicating a ground temperature increase after sea ice loss in accordance with the long-term tendency due to the global warming (Supplementary Figure 4a). On the other hand, when we looked at the relationship of interannual variations by conducting a detrend analysis, negative correlations appear over the eastern part of Eurasia, which indicates the occurrence of cold ground temperature anomalies after the sea ice loss, (Supplementary Figure 4b), while two stations near the Barents/Kara Seas still had positive correlations. Although the correlations are not statistically significant, these signals roughly correspond to our simulation results showing temperature response to sea ice loss (Fig. 2b and Fig. 3d in the main text).

## Supplementary References

1. Ogawa, F. *et al.* Evaluating Impacts of Recent Arctic Sea Ice Loss on the Northern Hemisphere Winter Climate Change. *Geophys. Res. Lett.* **45,** 3255–3263 (2018).
2. Hurrell, J. W., Hack, J. J., Shea, D., Caron, J. M. & Rosinski, J. A. A new sea surface temperature and sea ice boundary dataset for the Community Atmosphere Model. *J. Clim.,* **21,** 5145–5153 (2008).
3. Biskaborn, B. K. *et al.* Permafrost is warming at a global scale. *Nat. Commun.* **10,** 264 (2019).
